# Supplementary material for: Scanning-Probe Quantum Sensing of a Microwave and Static Magnetic Field Response of an On-Chip Superconducting Resonator
Source: Nano Lett. 2026 Aug 7;26(32):10636–42. doi: 10.1021/acs.nanolett.6c01668 (PMC13495936; doi:10.1021/acs.nanolett.6c01668)
Supplement: Supplementary file 1 [file nl6c01668_si_001.pdf]

Supporting Information for Scanning-Probe Quantum Sensing of a Microwave and Static Magnetic Field Response of an On-Chip Superconducting Resonator

Senlei Li[1], Jingcheng Zhou[1], Zelong Xiong[1], Hanyi Lu[2,1], and Hailong Wang[1*]

[1]School of Physics, Georgia Institute of Technology, Atlanta, Georgia 30332, USA
[2]Department of Physics, University of California, San Diego, La Jolla, California 92093, USA

[*]Corresponding author: hwang3021@gatech.edu

Section 1. Devices and methods

On-chip superconducting Nb coplanar waveguide resonators are prepared by standard photolithography, metal deposition, and lift-off processes. 200-nm-thick Nb is deposited by on-axis sputtering technique at room temperature on resonator patterns photolithographically defined on sapphire substrates. The base pressure of the sputtering chamber is below 2.0×10^{-8} Torr, and the Nb deposition rate is calibrated to be ~1.2 nm/s. Superconducting transition point of the Nb material is characterized to be ~9.1 K.

The presented scanning-probe NV magnetometry measurements are performed by a home-built confocal and a commercially available AFM operating in an optical cryostat (attocube systems Inc.). A diamond cantilever containing a single NV center is glued to a quartz tuning fork for force-feedback AFM operations, and a window on top of the cryostat provides optical access for NV sensing measurements. A sample holder is fixed onto a stack of piezo-based positioners and scanners to engage with the diamond cantilever and to perform scanning measurements. NV spin states are optically addressed by measuring NV PL using an avalanche photo diode. Microwave signals delivered to on-chip Au stripline are supplied by a Stanford Research Systems SG386 signal generator and external d.c. magnetic fields applied in scanning NV measurements are generated by a three-axis superconducting vector magnet. We implement pulsed microwave and optical sequences for NV Rabi oscillation measurements. The external magnetic field strength varies to tune the NV ESR frequency, and the input microwave driving frequency is set at f_{-} for Rabi measurements. Continuous green laser and microwave signals are applied to perform NV ODMR measurements.

Section 2. Simulations of microwave current and field distribution of an on-chip Nb resonator

We utilize 3D Ansys HFSS electromagnetic simulations to evaluate spatially dependent microwave magnetic field distribution of an on-chip Nb resonator. In our simulation model, we define a layer of superconducting material consisting of a coplanar center strip (signal line), a ground line, and an external microwave feedline in the same dimension and geometry of our prepared device. A sapphire substrate with a dielectric constant of ~9.4 is defined underneath the superconducting layer. We send a microwave stimulus at a frequency of ~2.865 GHz and numerically solve the 3D spatial distribution of microwave current density over the on-chip superconducting resonator. Figures S1(a)-S1(b) present HFSS simulated microwave current density J_x and J_y over the center strip and ground lines of a surveyed device area. One can see that directions of longitudinal (relative to the microwave center strip) microwave current J_x flowing in the signal and ground lines are opposite with each other. This can be qualitatively understood by the conservation of electrical charge currents in the superconducting circuit as the ground line essentially serves as a return path for currents flowing in the center strip. The transverse microwave current density J_y largely remains to be zero over the entire surveyed device area [Fig. S1(b)]. Based on the simulated current distribution, we can calculate the corresponding spatially dependent microwave Oersted field B_R (transverse to NV spin axis) generated by the superconducting resonator as presented in Fig. S1(c).

In our experiments, the magnitude of B_R transverse to NV spin axis can be deduced from NV Rabi oscillation frequency f_{Rabi} using the equation: $B_R = \frac{\sqrt{2} f_{Rabi}}{\gamma}$,[1] where γ = 2.8 MHz/G is the gyromagnetic ratio of an NV center. Our scanning-probe NV measurement results [Fig. S1(d)]

show satisfactory agreement with the theoretical calculations [Fig. S1(c)], demonstrating the capability of NV sensors in mapping local microwave magnetic field environment of an on-chip resonator device.

Section 3. NV imaging of superconducting vortices at different magnetic cooling field conditions

We perform scanning NV imaging measurements to visualize superconducting vortices formed in the Nb resonator at different external magnetic field cooling conditions. In these experiments, a small magnetic field B_z perpendicular to the device plane is applied during the field cooling process, and B_z is kept in the follow-up NV measurements. Figures S2(a)-S2(e) present a series of 2D maps of B_s measured at 2 K under different magnetic cooling field B_z (2.7 G, 0 G, −1 G, −1.3 G, and −6.3 G) normal to the sample surface. Due to the conservation of magnetic flux, number of vortices N over the surveyed sample area A and the magnetic cooling field B_z is expected to follow the equation: $N/A = B_z/\Phi$, where Φ is one magnetic flux quantum.[2] Figure S2(f) shows that N/A measured in the Nb center strip increases linearly with B_z with a slope of ~0.045 G^{-1} μm^{-2}. Magnetic flux quantum Φ is fitted to be ~22.2 G μm^2, in qualitative agreement with the theoretical value[2] of 20.7 G μm^2.

We introduce the autocorrelation analysis of magnetic field (B_s) maps to evaluate the periodicity of superconducting vortices. The magnetic field signal correlates with a spatially shifted copy of itself through the equation:[3,4] $\text{Autocorrelation}(\Delta x, \Delta y) = \sum_{x,y} B_s(x+\Delta x, y+\Delta y) B_s(x,y)$, where Δx and Δy are the spatial shift length. The autocorrelation analysis allows repeating features to be enhanced while suppressing random fluctuations of superconducting vortex patterns. As a result, characteristic periodic structures emerge in the autocorrelation map as shown in Fig. 3 in the main text.

We also measure the magnetic field dependent microwave transmission coefficient S_{21} of the patterned Nb resonator. The microwave transmission spectrum is recorded under different out-of-plane external magnetic field B_z (0 G, 40 G, 65 G, 80 G, and 100 G) at 2 K [Fig. S3(a)]. The resonance linewidth gradually broadens as B_z increases while the total transmission amplitude decreases.[5] Figure S3(b) shows the quality factor (Q factor) obtained as a function of B_z and superconducting vortex density N/A. The superconducting vortex density is estimated using the equation $N/A = B_z/\Phi$.

Section 4. Superconductivity-induced magnetic hysteresis of NV electron spin resonance transitions

We observe superconductivity-induced magnetic hysteresis of NV electron spin resonance (ESR) transitions at 2 K when the depinning force of superconducting vortices becomes stronger. Figures S4(a)-S4(b) present two NV ODMR maps measured during the forward and backward magnetic field sweeping processes. The NV center is positioned above the microwave center strip of the Nb resonator, and external magnetic field B_{ext} is applied along the NV spin direction. Discrete jumps of NV ESR lines occur at different magnetic field strengths when ramping up and ramping down the external magnetic field. Figure S4(c) summarizes the local magnetic field B_{NV} measured by the NV center as a function of B_{ext} at 2 K, showing the characteristic magnetic hysteresis behavior. Overall, B_{NV} exhibits a larger value when B_{ext} is swept from a high value back

to zero, which is attributed to persistent trapping and pinning of superconducting vortices during the decrease process of the magnetic field.

References

(1) Du, C.; van der Sar, T.; Zhou, T. X.; Upadhyaya, P.; Casola, F.; Zhang, H.; Onbasli, M. C.; Ross, C. A.; Walsworth, R. L.; Tserkovnyak, Y.; Yacoby, A. Control and Local Measurement of the Spin Chemical Potential in a Magnetic Insulator. *Science* **2017**, *357* (6347), 195–198.

(2) Thiel, L.; Rohner, D.; Ganzhorn, M.; Appel, P.; Neu, E.; Müller, B.; Kleiner, R.; Koelle, D.; Maletinsky, P. Quantitative Nanoscale Vortex Imaging Using a Cryogenic Quantum Magnetometer. *Nat. Nanotechnol.* **2016**, *11* (8), 677–681.

(3) Song, T.; Sun, Q.-C.; Anderson, E.; Wang, C.; Qian, J.; Taniguchi, T.; Watanabe, K.; McGuire, M. A.; Stöhr, R.; Xiao, D.; Cao, T.; Wrachtrup, J.; Xu, X. Direct Visualization of Magnetic Domains and Moiré Magnetism in Twisted 2D Magnets. *Science* **2021**, *374* (6571), 1140–1144.

(4) Li, S.; Sun, Z.; McLaughlin, N. J.; Sharmin, A.; Agarwal, N.; Huang, M.; Sung, S. H.; Lu, H.; Yan, S.; Lei, H.; Hovden, R.; Wang, H.; Chen, H.; Zhao, L.; Du, C. R. Observation of Stacking Engineered Magnetic Phase Transitions within Moiré Supercells of Twisted van Der Waals Magnets. *Nat. Commun.* **2024**, *15* (1), 5712.

(5) Song, C.; Heitmann, T. W.; DeFeo, M. P.; Yu, K.; McDermott, R.; Neeley, M.; Martinis, J. M.; Plourde, B. L. T. Microwave Response of Vortices in Superconducting Thin Films of Re and Al. *Phys. Rev. B* **2009**, *79* (17), 174512.

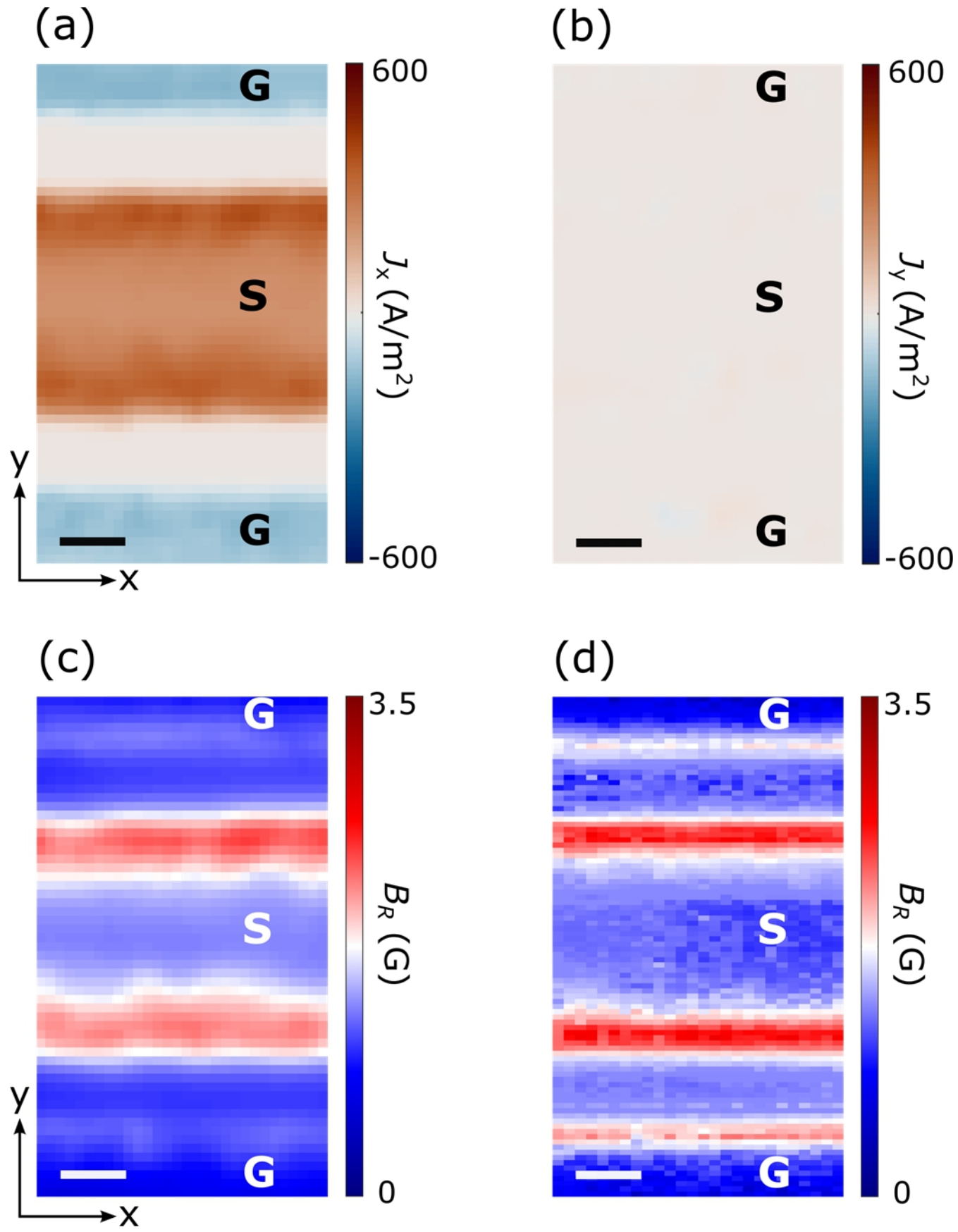

Figure S1. HFSS simulations of microwave electromagnetic response of a local ground-signal-ground (GSG) device area of an on-chip superconducting resonator. (a)-(b) Simulated longitudinal and transverse microwave current J_x and J_y distribution in an on-chip Nb resonator. (c) Spatial variations of microwave magnetic field B_R (transverse to NV spin axis) calculated from Figs. S1(a)-S1(b). (d) Scanning NV imaging of microwave magnetic field B_R distribution in the surveyed local GSG device area. The vertical NV-to-sample distance is ~500 nm, input microwave power is 10 dBm, and measurement temperature is 2 K. Scale bar is 6 μm in all figures.

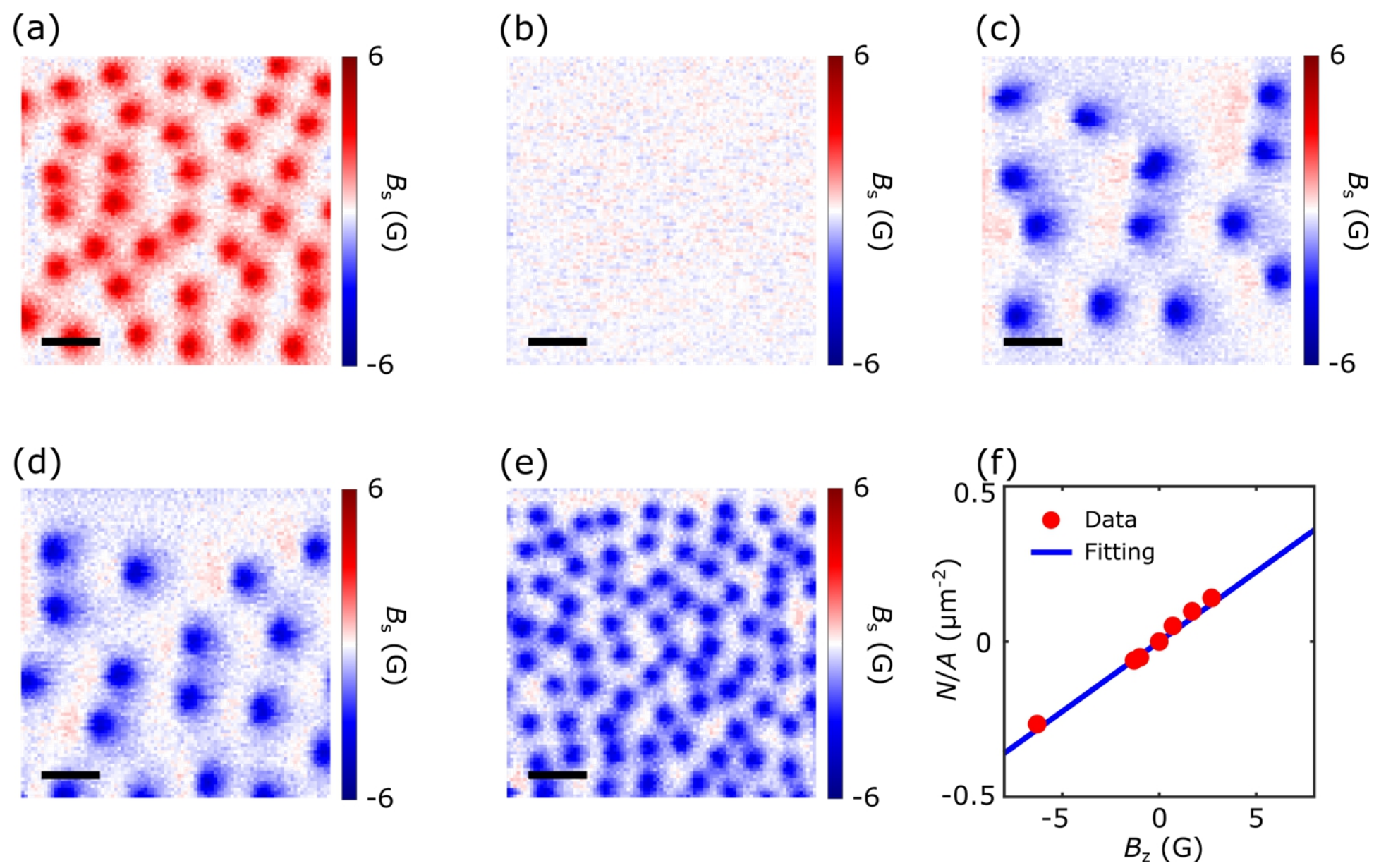

Figure S2. NV imaging of superconducting vortices under different magnetic cooling field strengths. (a)-(e) Field maps of superconducting vortices measured at magnetic cooling field B_z of 2.7 G [Fig. S2(a)], 0 G [Fig. S2(b)], −1 G [Fig. S2(c)], −1.3 G [Fig. S2(d)], and −6.3 G [Fig. S2(e)]. The scale bar is 2 μm. (f) Red points: Vortex density N/A as a function of magnetic cooling field B_z. Blue line: a linear fitting of the experimental data gives a slope of ~0.045 G^{-1} μm^{-2} with about zero interception in *y*-axis. The presented data is measured at 2 K with a vertical NV-to-sample distance of ~100 nm.

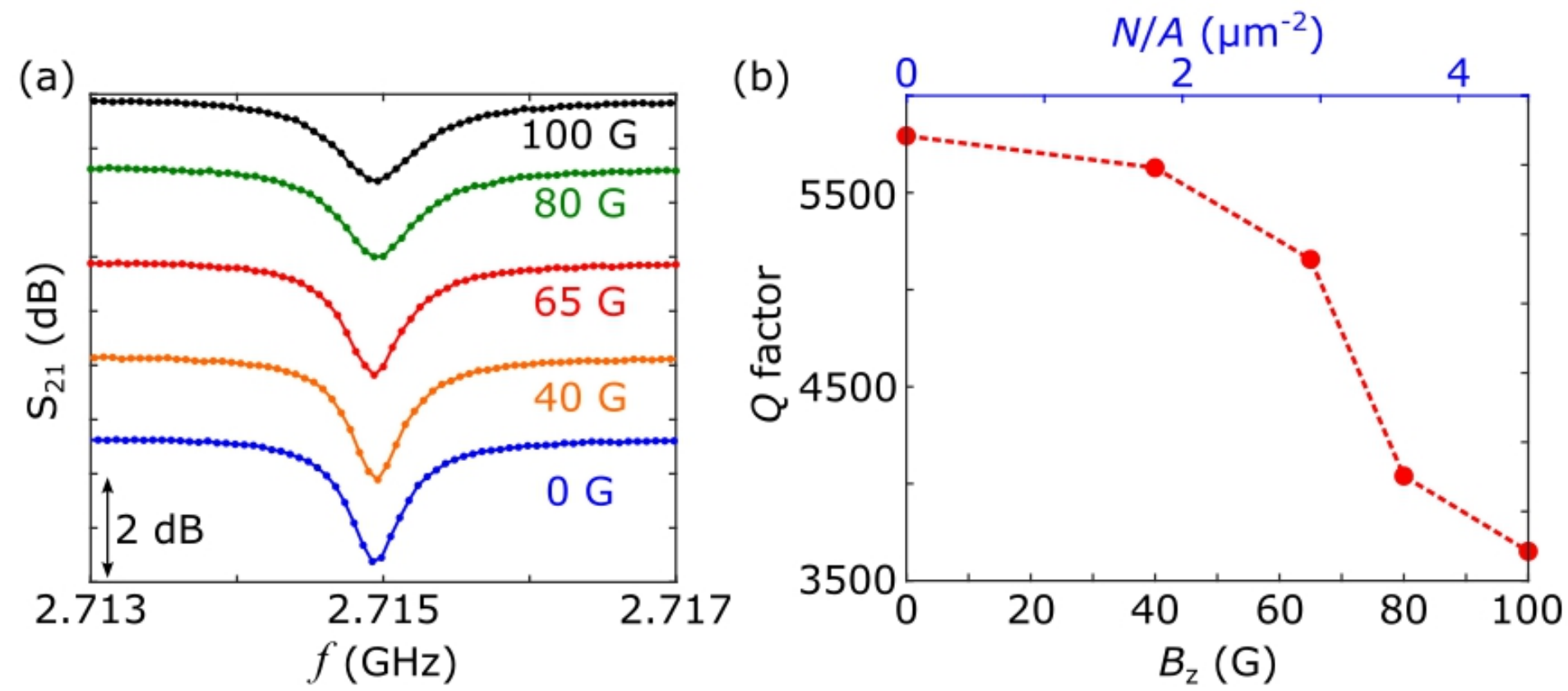

Figure S3. Field dependence of Nb resonator microwave transmission. (a) Microwave transmission spectra of the Nb superconducting resonator mode measured under different out-of-plane external magnetic field B_z at 2 K. (b) Quality factor Q obtained as a function of B_z (bottom) and estimated superconducting vortex density N/A (top) at 2 K.

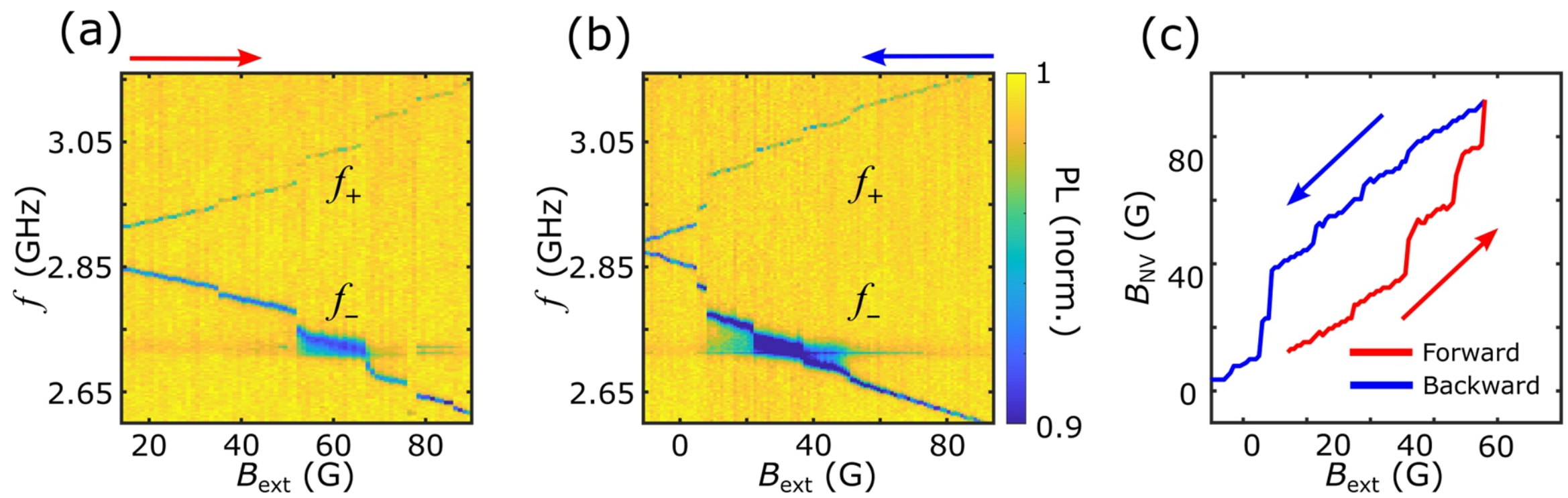

Figure S4. Superconductivity-induced magnetic hysteresis of NV ESR transitions. (a)-(b) NV ODMR maps showing normalized PL intensity as a function of external field B_{ext} and microwave frequency f during the forward (a) and backward (b) magnetic field sweeping processes. B_{ext} is applied along the local NV spin direction, and the measurement temperature is 2 K. (c) Local magnetic field B_{NV} measured by the NV center showing magnetic hysteresis behavior. The red and blue arrows represent ramping up and ramping down processes of the external magnetic field.
